# Radiological and Clinical Features and Outcomes of Patients with Primary Pulmonary Salivary Gland-Type Tumors

**DOI:** 10.1155/2019/1475024

**Published:** 2019-04-01

**Authors:** Xiaoyu Han, Jianchu Zhang, Jun Fan, Yukun Cao, Jin Gu, Heshui Shi

**Affiliations:** ^1^Department of Radiology, Union Hospital, Tongji Medical College, Huazhong University of Science and Technology, 1277 Jiefang Rd, Wuhan, Hubei Province 430022, China; ^2^Department of Respiratory Medicine, Union Hospital, Tongji Medical Collage, Huazhong University of Science and Technology, 1277 Jiefang Rd, Wuhan, Hubei Province 430022, China; ^3^Department of Pathology, Union Hospital, Tongji Medical Collage, Huazhong University of Science and Technology, 1277 Jiefang Rd, Wuhan, Hubei Province 430022, China

## Abstract

**Aim:**

To analyze the radiological, clinical, and prognostic features of primary pulmonary salivary gland-type tumors (SGTs) and improve their diagnosis.

**Materials and Methods:**

We retrospectively collected clinical and pathological data for 32 SGT cases confirmed by pathology and analyzed their radiological features, clinical presentations, and treatment outcomes.

**Results:**

Mucoepidermoid carcinoma (MEC) was more likely to occur in younger patients than was adenoid cystic carcinoma (ACC) (35 ± 15 years vs 48 ± 16 years, *p*=0.038). MEC was equally distributed between both sexes, whereas ACC was more frequent in females (66.7%). The main presenting symptom of SGT was cough (56.3%), followed by dyspnea (40.6%), associated with the tumor location. ACC more frequently involved the trachea or main bronchus (86.7% vs 25.0%, *p*=0.001) and more commonly presented as lobulated or circumferential thickening than MEC (93.3% vs 37.5%, *p*=0.002). MEC more frequently presented as obvious enhancement than ACC (68.8% vs 31.3%, *p*=0.001). CT findings suggestive of airway obstructive disease were more likely to be observed with MEC than ACC (73.3% vs 25.0%; *p*=0.021). The SUVmax in 8 of 10 patients with PET/CT data exceeded 2.2 but was less than 6.0. The overall survival (OS) at 3 and 5 years was 90.9% and 72.2% in all patients, respectively. Tumor-node-metastasis (TNM) stage, surgery, and patient age were associated with OS (*p* ≤ 0.001, *p*=0.001, and *p*=0.001, respectively).

**Conclusion:**

SGTs commonly occur in patients at a young age and are associated with weak invasive features and a good prognosis. The predominant site and CT characteristics are significantly different between ACC and MEC.

## 1. Introduction

Primary pulmonary salivary gland-type tumors (SGTs) account for approximately 0.1% to 0.2% of all lung cancers [1–5]. SGTs are rare, have low malignancy, and are thought to originate from the submucosal glands of central airways [1–3]. These tumors include adenoid cystic carcinomas (ACCs), mucoepidermoid carcinomas (MECs), epithelial-myoepithelial carcinomas (EMECs), and pleomorphic adenomas (PAs) according to the 2015 World Health Organization Classification of Tumors of the Lung [6]. MEC and ACC are the two most common histological subtypes of SGTs, and other subtypes are very rare [1, 3, 5].

Imaging, especially computed tomography (CT), is the main technique used to detect suspected airway lesions. Some CT features of both ACC and MEC have been discussed in the literature [3–5, 7–11]. Although MEC and ACC are both subtypes of SGTs, their clinical and CT characteristics differ. Most MECs are observed in segmental or lobar bronchi and appear as sharply well-defined, either ovoid or lobulated, intraluminal nodules that adapt to the branching features of the airways [3]. Lymph node metastasis is rare [5, 11]. ACCs are more common in the lower trachea or mainstem bronchi, while peripheral or segmental localization is uncommon [1, 3, 4]. ACCs have a significant tendency toward submucosal extension within the airway with infiltrated growth and commonly present with circumferential thickening or intraluminal and extraluminal extension [5, 12].

To our knowledge, only a few studies have focused on the CT findings of SGT, and the results are controversial [3–5, 7–11]. In addition, differences in CT findings and clinical features between MEC and ACC remain undefined because of the rarity of these two tumor types. The present study collected images and clinical records of a relatively large series of patients with SGT to summarize the imaging findings and clinical features. The treatments and outcomes of these patients were also explored.

## 2. Materials and Methods

### 2.1. Clinical Data

This retrospective study was approved by the local institutional review board. We collected clinical and pathological data from 32 patients with primary pulmonary SGT confirmed by operation, bronchofiberscopy, or CT-guided percutaneous lung biopsy in Wuhan Union Hospital between October 2009 and November 2017. Of 51 patients, 19 were excluded due to a lack of available pretreatment CT images. Patient clinical characteristics, including age, sex, history of smoking, histopathology, tumor size, nodal involvement, and staging and distant metastasis, were recorded. A diagnosis of MEC, ACC, or EMC was made according to the World Health Organization Classification of Tumors of the Lung [6]. MEC was graded in line with the algorithm proposed by Yousem and Hochholzer [13]. Grading of ACC and EMC was not performed. Staging was performed in compliance with the tumor-node-metastasis (TNM) staging system of the Union for International Cancer Control (8th edition). Follow-up was conducted via telephone interviews. In total, 32 patients (16 with MEC, 15 with ACC, and 1 with EMC) were recruited.

### 2.2. Imaging

All 32 patients underwent nonenhanced CT scanning, 26 patients underwent enhanced CT scanning, six patients underwent only nonenhanced CT, eight patients underwent X-ray, 10 patients underwent PET/CT scanning, and two underwent magnetic resonance imaging (MRI). All patients were scanned with a multislice spiral CT (Somatom Definition AS+, Siemens Healthineer Germany) of the following parameters: detector collimation width 128 × 1; tube voltage 120 kV; pitch 1.2; image thickness 5 mm; image interval 5 mm; and matrix 512 × 512. The tube current was quickly adjusted to the automatic exposure control system (care dose 4D). Thin-slice reconstruction (1.5 mm) and image postprocessing were performed after scanning. The nonionic iodine contrast agent (60–80 mL iohexol 350 mg/mL, Beilu Pharmaceutical Co., Ltd; Beijing, China) had been applied to 26 patients with intravenous injection of the elbow, and the dose was 3 mL/s.

All images were evaluated by two experienced chest radiologists who were unaware of the clinical and histologic findings but were aware of the presence of the tumors. After they performed separate evaluation, the differences were resolved by consensus. For each CT scan, the following data were recorded on an Excel spreadsheet file: (1) location: central location—tumor located in the segmental bronchi or above and peripheral location—tumor located in the subsegmental bronchi or more distal airway; (2) maximum diameter of the lesion (in centimeters) evaluated on the multiplanar reconstructions (MPRs) with a soft tissue window; (3) shape: indicated as circumferential airway thickening, lobulated, or round; (4) margin definition: well defined or poorly defined; (5) heterogeneity: evaluated in the tissue window and indicated as homogeneous or heterogeneous; (6) lymphadenopathy: indicated as hilar or mediastinal lymphadenopathy with short-axis diameter greater than 1 cm; (7) degree of enhancement: “mild” = 0∼20 HU, “moderate” = 20∼40 HU, and “marked” > 40 HU [11]; (8) obstructive changes: presence or absence of obstructive changes; and (9) distant metastasis: presence or absence of distant metastasis.

PET/CT experiments were carried out using the Discovery LS PET/CT system (GE Medical System). Ten patients were fasted for at least 6 hours before the scans. Before intravenous infusion of 5.5 MBq/kg of ^18^F-FDG, the blood glucose concentration was less than 6.6 mmol/l. Imaging acquisition was performed 1 h after ^18^F-FDG administration. The CT was attenuated with the following parameters: 120 kV, 80 mA, and 4.25 mm collimation. A PET scan was then performed from the head to the upper leg in a 2-dimensional mode, per bed position for 3 minutes. The examination of 6–8 beds typically depended on the height of the patient. The ordered set expectation maximization algorithm method was used to reconstruct PET data. The PET data were subjected to attenuation correction and anatomical positioning using CT images. The evaluation of coregistered images was displayed on a Xeleris Workstation (GE Healthcare System).

### 2.3. Statistical Analyses

Statistical analyses of all data were performed with SPSS software (SPSS 21.0 for Windows, IBM, Chicago, IL, USA). Normality of all continuous data was checked using the Kolmogorov–Smirnov test. Normally and nonnormally distributed data and categorical variables were expressed as the mean ± standard deviation, median (interquartile range), and frequencies (percentages), respectively. Categorical variables were presented as counts and percentages, and comparisons were conducted using Fisher's exact tests. Overall survival (OS) was calculated from the date of the initial pathological diagnosis to the date of death or last follow-up. Kaplan–Meier survival curves were used to estimate survival, and the log-rank test was used to assess differences between groups. All *p* values were two-sided. A *p* value of <0.05 was considered significant. No adjustment for multiple comparisons was performed.

## 3. Results

### 3.1. Clinical Features

The average age of the patients with SGT was 41 ± 16 years (range, 18–68 years); MEC was more likely to occur in younger patients than ACC (35 ± 15 years vs 48 ± 16 years; *p*=0.038). MEC was equally distributed between both sexes, whereas ACC was more frequent in females (66.7%). There was no significant difference in symptoms between MEC and ACC (Table 1). Seven of the 32 patients had been mistreated for other diseases (pneumonia = 1, tuberculosis = 2, and asthma = 4). One patient received antituberculosis treatment for 9 months. Among the group data, 14 patients with MEC (87.5%) presented with stage I or II disease, and 11 patients with ACC (73.3%) presented with stage I or II disease (Table 1). There were 14 low-grade and one high-grade MEC tumor, and one did not have a defined grade.

### 3.2. Images

Lesions were found in the central (25/32, 78.1%) and peripheral lung (7/32, 25.9%). ACC more frequently involved the trachea or main bronchus than MEC (86.7% vs 25.0%, *p*=0.001).

**Figure 1:**
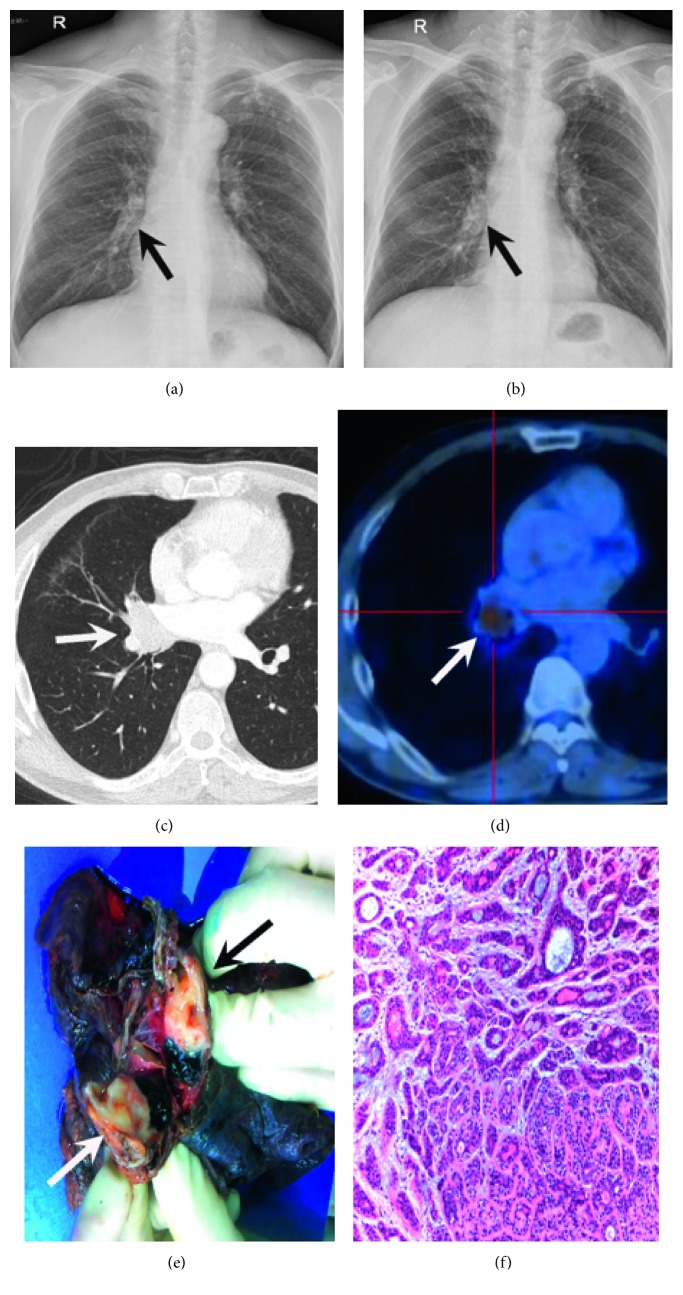
A 54-year-old man with adenoid cystic carcinoma. (a) Frontal chest radiograph showing some fullness in the right suprahilar region (arrow), and (b) greater fullness was observed (arrow) after two years. (c) Axial chest CT image showing a 2.2 × 3.2 cm mass (arrow) in the right middle lobe obstructing the right bronchus intermedius. (d) PET/CT image showing intense nonhomogeneous ^18^F-FDG uptake in the tumor (arrow). The maximum standardized uptake value was 5.3. (e) Gross pathologic specimen showing a yellow-white mass consisting of an intraluminal lesion (arrow). (f) High-magnification photomicrograph showing that the tumor is composed mainly of mucus-secreting cells arranged in a funicular and cribriform pattern within the gland (*H* and *E* ×100).

Eight patients had undergone X-ray before any treatment. Four patients were negative on X-ray—one lesion occurred in the segmental bronchi and showed some fullness in the right suprahilar region (Figures 1(a) and 1(b)), two peripheral lesions presented as smooth round nodules, and the remaining lesion was only observed as distal obstructive atelectasis.

**Figure 2:**
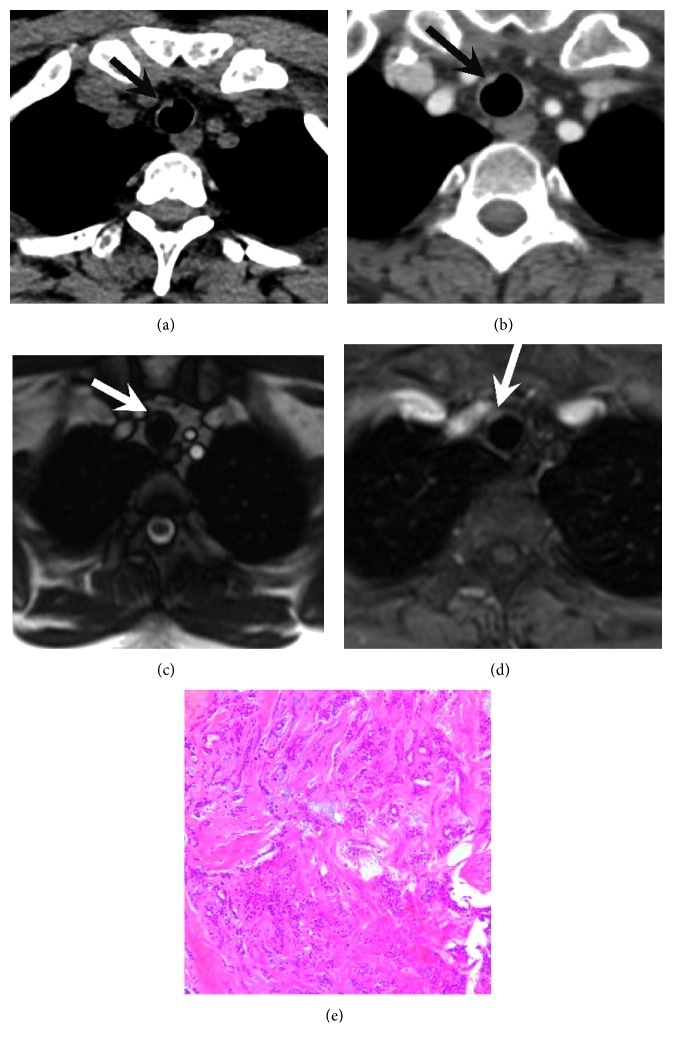
A 36-year-old woman with adenoid cystic carcinoma. (a) CT and (b) contrast CT images demonstrating a poorly defined, tiny nodule in the trachea (arrow). (c) MR and (d) contrast MR images clearly showing the location of the tumor (arrow) in the trachea and surrounding tissues. (e) High-magnification photomicrograph showing that the tumor consists of uniform compact cells in a cribriform (glandular) pattern with little atypism or mitotic activity (*H* and *E* ×100).

**Figure 3:**
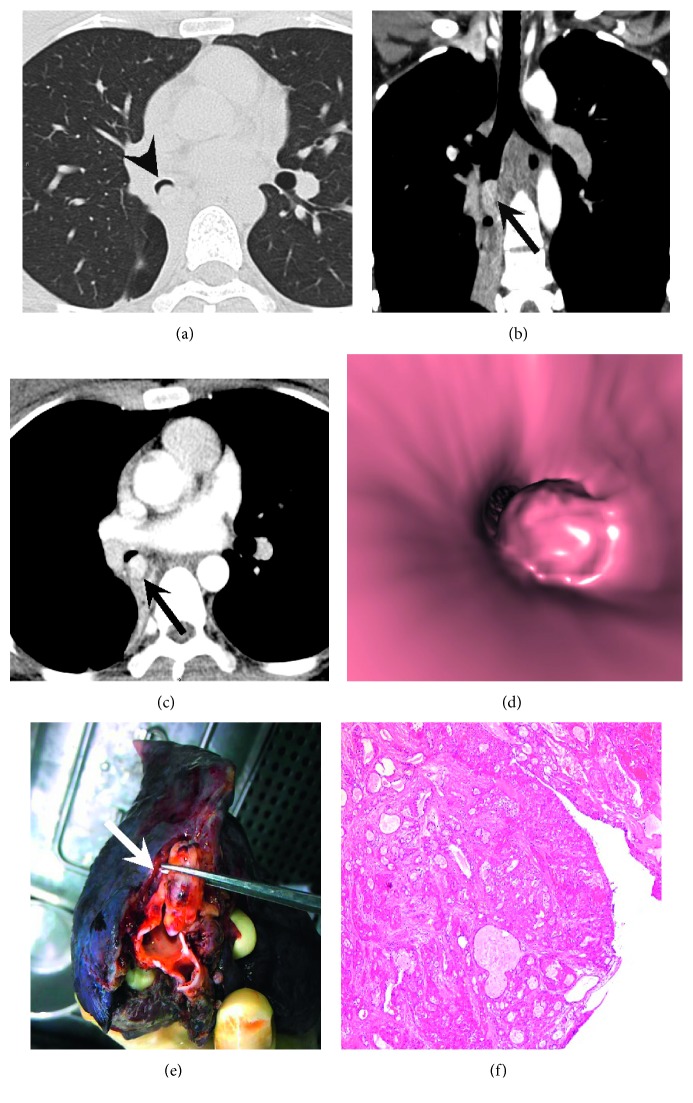
A 22-year-old woman with low-grade mucoepidermoid carcinoma. (a) Unenhanced transaxial CT images showing a well-defined mass in the right lower bronchus with a crescent-shaped gap around the tumor (“air crescent sign”) (arrowheads). (b) Coronal oblique reconstruction image of contrast-enhanced CT shows an endobronchial long oval-like nodule (arrow); the direction of the longest diameter is parallel to the branching pattern with associated atelectasis of the right lower lobe. (c) In the contrast-enhanced transaxial CT, the mass shows marked enhancement (arrow). (d) Virtual bronchoscopy (VB) showing an intraluminal nodule wide base. (e) Gross pathologic specimen showing an intraluminal yellow-white nodule in the right lower bronchus (arrow). (f) Photomicrograph showing that the tumor consists of cells with little atypism or mitotic activity (*H* and *E* ×40).

Of the 10 patients with the central MEC, intraluminal (8/10, 80%) and extraluminal (2/10, 20%) extensions were observed on CT. Among the 14 patients with central ACC, intraluminal extensions (6/14, 42.9%), infiltrative wall-thickening lesions with (2/14, 14.3%) or without (3/14, 21.4%) focal nodules (Figure 2), and extraluminal extensions (3/14, 21.4%) were identified. Twenty patients showed a crescent-shaped gap around the tumor (“air crescent sign;” Figure 3(a)). Tumors were round to oval (10/32, 31.3%), lobulated (16/32, 50%), or had circumferential thickening (6/32, 18.6%). The MECs were mainly round to oval (10/16, 62.5%), but most ACCs were lobulated (Figure 1(c)) or had circumferential thickening (14/15, 93.3%; *p*=0.002). The average maximal tumor size was 2.2 ± 1.3 cm overall, and there was no difference in tumor size between MEC and ACC. However, the median size of peripheral tumors was significantly larger than that of central tumors (3.0 vs 1.5 cm; *p*=0.041). Obvious enhancement was more commonly observed in MEC than in ACC (66.7% vs 9.1%, *p*=0.001) (Figures 3(b) and 3(c)). Associated CT findings suggestive of airway obstruction disease were found in 16 patients (MEC = 12 and ACC = 4) and included distal bronchial regions with postobstructive pneumonia (7/16, 43.8%), subsegmental atelectasis (5/16, 31.3%), mucoid impaction (4/16, 25%), and obstructive emphysema (1/16, 6.3%). Mediastinal or hilar lymph node enlargement was observed in both MEC (2/16, 12.5%) and ACC (4/15, 26.7%) (Table 2).

Of the two patients with ACC who had undergone MR examination, both were characterized by midrange T1WI signals and somewhat high T2WI signals with mild enhancement (Figures 2(c) and 2(d)).

Ten patients had undergone FDG PET/CT, and the median maximum standardized uptake value (SUVmax) was 2.8 (range, 0–5.3). MECs (*n* = 4) showed heterogeneous uptake in two patients and homogeneous uptake in one patient, and the SUVmax was 2.7 (range, 0–4.2). ACCs (*n* = 6) showed heterogeneous FDG uptake in four patients (Figure 1(d)) and homogeneous uptake in one patient, and the median SUVmax of ACC was 3.1 (range, 0–5.3). No hilar or mediastinal nodal metastasis was found by PET/CT.

### 3.3. Treatment and Follow-Up

Most SGT patients were treated with surgery (24/32, 75%) regardless of tumor histology (*p*=1.000). The most common procedures for SGT patients were tracheal resection (40.6%), followed by lobectomy (31.3%) and pulmonary wedge resection (3.1%). In addition, a positive resection margin was more frequently observed in ACC than in MEC (60% vs 7.7%, *p*=0.019). Adjuvant treatment in the form of radiotherapy (*n* = 18) or chemotherapy (*n* = 4) was given to 22 patients.

**Figure 4:**
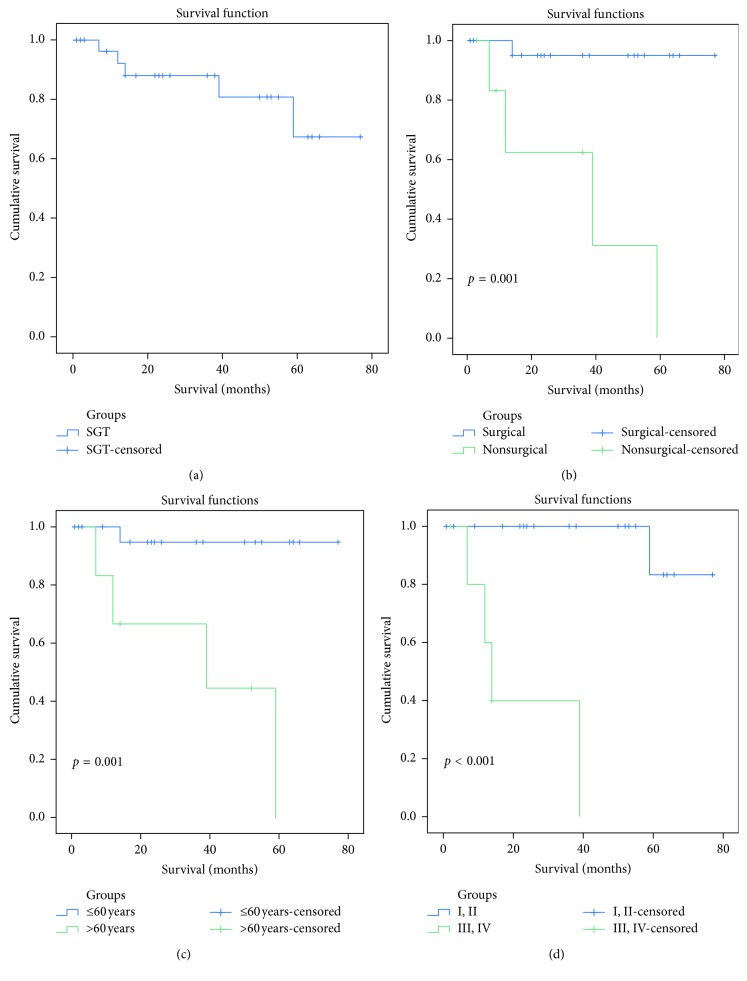
Kaplan–Meier overall survival (OS) curves of all patients (a), patients with or without surgery (b), patients of different ages (c), and patients with various TNM stages (d).

Survival information was available for 31 of 32 patients with SGT (96.9%). The median follow-up duration was 49 months (range, 1–96 months). The OS at 3 and 5 years was 90.9% and 72.2% in all patients, respectively (Figure 4(a)). No significant difference was found between patients with ACC and MEC regarding OS (*p*=0.642). TNM stage, surgery, and age were found to be associated with OS (*p* < 0.001, *p*=0.001, and *p*=0.001, respectively) (Figures 4(b)–4(d)). Tumor recurrence was identified in three patients with ACC (all patients had positive resection margin) at an average of 65.3 months (range, 36–95 months). One of those patients had relapsed for the second time, and the time interval to the first recurrence was 10 years according to his medical history. Metastasis was observed in two patients (MEC = 1 and ACC = 1). No significant difference was found between the two groups regarding recurrence or metastasis.

## 4. Discussion

Currently, the images and clinical features of SGTs remain unclear because of the rarity of these tumors. Our study revealed that patients with SGT commonly had a good prognosis, but the disease easily leads to a misdiagnosis due to its rarity and few characteristic clinical features. CT is a very useful tool to detect lesions early and to help in differentiating ACC from MEC.

MEC and ACC are reported as the most common subtypes of SGT of the lung [1, 2, 11, 14], and ACC is reported as the second most common tumor of the trachea after squamous cell carcinoma [4, 8, 15]. In our series, the most common histologic subtype was MEC, followed by ACC. Clinically, MEC in the present study was equally distributed between both sexes, whereas ACC was more frequent in females, consistent with the report by Molina et al. [5]. Generally, the mean age of patients with MEC was lower than that of patients with ACC, and 50% of patients with MEC were younger than 30 years [5, 16, 17]. Patients commonly present with symptoms of bronchial obstruction, and the two most frequent two symptoms are cough and dyspnea. The tumor location generally accounts for the frequency of symptoms, and therefore, the majority of peripheral tumors are asymptomatic and detected incidentally [5, 16]. It is widely believed that smoking is not a risk factor for these two tumor types [1, 3, 5]. The findings in our study were in agreement with the above reports. Because these tumors lack specific clinical characteristics and mimic other benign diseases, such as tuberculosis and asthma, which also occur in young people [15], seven of our patients (21.9%) had been mistreated for other diseases (pneumonia = 1, tuberculosis = 2, and asthma = 4). Thus, awareness of SGTs should be increased to lower the rate of misdiagnosis. High-grade tumors have been reported to account for 20–25% of MEC [7, 18]. In the present study, only two MEC patients were diagnosed with high-grade tumors (12.5%); however, the grading for ACC and EMC tumors is still not well defined [2].

The value of X-ray in the diagnosis of SGT was very limited for the following two reasons: First, SGTs were mostly located in the central airway and are easily overlapped by mediastinal tissue and bone shadow. Second, peripheral SGTs usually appeared as pulmonary nodules or masses without any characteristic features.

Generally, CT scans are effective and noninvasive methods that can be used to detect suspected airway or lung lesions, particularly in the upper airway. SGTs originate from the submucosal bronchial glands; these tumors are often central lesions. Consequently, SGTs, especially ACC, develop mainly in the central airway and are rarely found in the peripheral lung [2, 3, 5]. In our series, the majority of tumors were centrally located (78.1%). ACCs involved more frequently larger airways (trachea and main bronchus) than MECs, consistent with previous studies [1, 3]. Twenty-five patients had central lesions, and more than 68% of these lesions manifested as endobronchial nodules. As mentioned in previous literature [7, 11], some evidence indicate that tumors originate from endobronchial sources: First, obstructive signs and “air crescents” (Figure 3(a)) were caused by bronchi which are partially or completely blocked by an endobronchial tumor. Second, the longest diameter of these tumors was commonly parallel to the branching pattern (Figure 3(b)).

Previous studies found the median size of ACC was larger than that of MEC [3, 5]. However, no significant difference was found between the median size of MEC and ACC in our patients. Notably, the median size of peripheral tumors was significantly larger than that of central tumors. The following two reasons may explain this phenomenon. First, central lesions more often cause airway obstruction or initiate symptoms in the early stage than peripheral lesions. As a result, patients with central tumors tend to be diagnosed before the tumor becomes large. Second, the airway restricts the growth of central tumors but not peripheral tumors. Most MECs in our patients appeared as well-defined masses with oval or round shapes and smooth margins, similar to several previous reports [2, 7, 11]. In contrast, most ACCs in the present study manifested as lobulated lesions or circumferential thickening due to infiltrative growth, indicating stronger invasion than MECs.

In addition, the prevalence of calcification of MECs varies from 9.1% to 50% according to previous reports [7, 10, 11]. In the present study, visible sand-like or patchy calcification was found in three patients (18.8%) on nonenhanced CT. Intratumor calcification may be caused by insufficient absorption of mucus [11]. There was no calcification observed in ACC, and there are no related reports in the literature. In the present study, most MEC patients showed marked enhancement (66.7%), while ACC showed mainly mild or moderate enhancement (90.1%). Kim and Cheng found that MEC commonly showed slight enhancement [7, 11]. However, among the five patients reported by Ishizumi et al., four patients (80%) showed marked enhancement [9]. Such a distinction may be due to the diversity and complicated demographic characteristics of the patients and bias from a small cohort. On the other hand, Ishizumi et al. [9] found that most of the mucus of MECs was extensively distributed in blood vessels, which may have contributed to the high enhancement of MEC [7]. Generally, lymph node or distant metastases are uncommon in SGTs, indicating mildly aggressive features [3, 11]. In the present study, the majority of SGTs showed no suspicious lymph node or intrathoracic metastasis.

Both CT and MRI are useful for SGT diagnosis, with MRI being superior at determining the extent of disease and differentiating recurrence from postradiotherapy fibrosis [19, 20]. In our two ACC patients, MRI was very useful for pretreatment assessment and evaluating therapeutic response in terms of possible disease recurrence and radiation-induced changes. Regarding the degree of FDG uptake, Elnayal et al. [3] reported that FDG uptake of the primary tumor is greater in ACC than in MEC and EMC. In addition, Jeong et al. [21] found that ACC and MEC show variable amounts and patterns of FDG uptake within tumors, and high-grade tumors have greater FDG uptake. In our study, primary tumors had a somewhat high median FDG uptake (<6.0). However, no significant difference in FDG uptake was found between ACC and MEC. Such a difference in findings might be due to the small cohort of our patients.

Surgical intervention is considered the main treatment option for SGT, and complete surgical resection is associated with favorable long-term outcomes [2]. The 5-year survival rate of patients with primary SGT of the lung in our study was 72.2%, which was substantially better than the prognosis for patients with more common lung cancers, such as adenocarcinoma (with a 5-year survival rate of 20%) and squamous cell carcinoma (with a 5-year survival rate of 17%) as reported [22]. No significant difference was found between the ACC and MEC groups regarding OS. However, in a large-sample study, Kumar et al. demonstrated that patients with ACC were significantly worse than patients with MEC only after 5 years [23]. Such a difference may be due to our short follow-up period (49 months). A total of 26 patients in our group are still being followed for further outcomes. Moreover, TNM stage, surgery, and age were associated with OS.

However, a good prognosis does not always indicate an indolent tumor. Azar et al. [12] suggested that local recurrence of ACC occurs at an average of 51 months after the primary treatment. Chin et al. [24] found that tumors may recur over as long as 30 years. In our series, three of the ACC patients had local recurrence at an average of 65.3 months (range, 36–95 months) after surgery. One of the patients had relapsed for the second time, and the time interval to the first recurrence was 10 years. One possible explanation for recurrence may be that the extent of submucosal infiltration of ACC is often difficult to assess before and during surgery. Additionally, complete resection may be very difficult to achieve, emphasizing the need to initiate radiation therapy in patients with positive resection margins at the time of surgery or for palliative treatment.

In conclusion, SGT occurs mainly in younger people and is associated with a substantially better prognosis than common lung cancers. The differences in CT features between MEC and ACC were statistically significant. Nevertheless, our study is limited by its retrospective design and small size, and further prospective studies and larger series can help in obtaining more solid conclusions.

## Figures and Tables

**Table 1:** Clinical features of SGT.

Variable	No. of patients (%)				*p* ^$^
	All patients	EMC	MEC	ACC	
Age (years)	41 ± 16	43	35 ± 15	48 ± 16	0.038

Sex					0.285
Male	14 (43.8)	0	9 (56.3)	5 (33.3)	
Female	18 (56.3)	1	7 (43.7)	10 (66.7)	

Clinical symptom					
Cough	17 (53.1)	0	10 (62.5)	7 (46.7)	0.479
Dyspnea	13 (40.6)	1	4 (25)	8 (53.3)	0.285
Chest pain	5 (15.6)	0	5 (31.3)	0 (0)	0.043
Hoarseness	1(3.1)	0	0(0)	1(6.7)	1.000
Hemoptysis	2 (6.3)	0	2 (12.5)	0 (0)	1.000
Blood-stained sputum	1 (3.1)	0	0 (0)	1 (6.7)	1.000
Asymptomatic	3 (9.4)	0	2 (12.5)	1 (6.7)	1.000
Dysphagia	2 (6.3)	0	0 (0)	2 (14.3)	1.000

Smoker					1.000
No	30 (93.8)	1	16 (100)	13 (86.7)	
Yes	2 (6.3)	0	0 (0)	2 (13.3)	

TNM stage					
I	22 (68.8)	1	11 (68.8)	10 (66.7)	1.000
II	4 (12.5)	0	3 (18.8)	1 (6.7)	1.000
III	4 (12.5)	0	1 (6.3)	3 (20)	0.333
IV	2 (12.5)	0	1 (6.3)	1 (6.7)	1.000

Treatment		0			
Surgical	24 (75)	1	13 (81.2)	10 (66.7)	0.433
Tracheal resection	12 (37.5)	1	3 (23.1)	8 (80)	0.066
Lobectomy	11 (34.4)	0	9 (69.2)	2 (20)	0.023
Wedge lobectomy	1 (3.1)	0	1 (7.8)	0 (0)	1.000
Adjuvant therapy					
No	10 (31.3)	1	8 (50)	1 (6.7)	0.015
Chemotherapy	4 (12.5)	0	2 (12.5)	2 (13.3)	1.000
Radiotherapy	18 (56.3)	0	6 (40)	12 (75)	0.029

Resection margin					0.019
Negative	17 (53.1)	1	12 (92.3)	4 (40)	
Positive	7 (21.9)	0	1 (7.7)	6 (60)	

MEC, mucoepidermoid carcinoma; ACC, adenoid cystic carcinoma; TNM, tumor-node-metastasis. ^$^*p* values for comparisons between MEC and ACC for each variable.

**Table 2:** CT features of SGT.

Variable	No. of patients (%)				*p* ^$^
	All patients	EMC	MEC	ACC	
Location					0.001
Trachea, main bronchi	18 (56.3)	1	4 (25)	13 (86.7)	
Other locations	14 (43.8)	0	12 (75)	2 (13.3)	

Median size^#^ (cm, range)	2.0 (18–68)	1.1	2.3 (18–66)	1.7 (22–68)	0.912

Homogeneous					0.149
Yes	19 (59.4)	1	7 (43.8)	11 (73.3)	
No	13 (40.6)	0	9 (56.3)	4 (26.7)	

Margin definition					0.073
Well defined	19 (59.4)	1	12 (75)	6 (40)	
Poorly defined	13 (40.6)	0	4 (25)	9 (60)	

Shape^*∗*^					0.002
1	20 (62.5)	0	6 (37.5)	14 (93.3)	
2	12 (37.5)	1	10 (63.5)	1 (6.7)	

Enhancement degree					0.001
Mild or moderate	15 (46.9)	—	5 (33.3)	10 (90.1)	
Evident	11 (34.4)	—	10 (66.7)	1 (9.9)	

Lymphadenopathy					1.000
Yes	6 (18.8)	0	2 (14.3)	4 (26.7)	
No	26 (81.3)	1	14 (87.5)	11 (73.3)	

Suspected metastasis					1.000
Yes	2 (6.3)	0	1 (6.3)	1 (6.7)	
No	30 (93.8)	1	15 (93.7)	14 (93.3)	

Airway obstruction					0.012
Yes	16 (50)	0	12 (75)	4 (26.7)	
No	16 (50)	1	4 (25)	11 (73.3)	

MEC, mucoepidermoid carcinoma; ACC, adenoid cystic carcinoma. ^#^the largest diameter of the tumor. ^*∗*^Shape type: 1, circumferential thickening or lobulated; 2, round or oval. ^$^*p* values for comparisons between MEC and ACC for each variable.

## Data Availability

The data used to support the findings of this study are available from the corresponding author upon request.
